# Assessment of the Environmental Impact of a Car Tire throughout Its Lifecycle Using the LCA Method

**DOI:** 10.3390/ma12244177

**Published:** 2019-12-12

**Authors:** Katarzyna Piotrowska, Weronika Kruszelnicka, Patrycja Bałdowska-Witos, Robert Kasner, Jacek Rudnicki, Andrzej Tomporowski, Józef Flizikowski, Marek Opielak

**Affiliations:** 1 Faculty of Mechanical Engineering, Lublin University of Technology, 20-618 Lublin, Poland; k.piotrowska@pollub.pl (K.P.); m.opielak@pollub.pl (M.O.); 2 Department of Technical Systems Engineering, Faculty of Mechanical Engineering, University of Science and Technology in Bydgoszcz, 85-796 Bydgoszcz, Poland; patrycja.baldowska-witos@utp.edu.pl (P.B.-W.); robert.kasner@gmail.com (R.K.); a.tomporowski@utp.edu.pl (A.T.); fliz@utp.edu.pl (J.F.); 3 Faculty of Ocean Engineering and Ship Technology, Gdańsk University of Technology, 80-980 Gdańsk, Poland; jacekrud@pg.edu.pl

**Keywords:** rubber, car tire, recycling, LCA, environmental impact

## Abstract

There are numerous threats to the natural environment that pose a significant risk both to the environment and to human health, including car tires. Thus, there is a need to determine the impact of the life cycle of car tires on the environment, starting with the processes of raw materials acquisition, production, and ending with end-of-life management. Therefore, the authors of this study chose to do research on passenger car tires (size: P205/55/R16). As part of the research, the life cycle assessment (LCA) of traditional car tires was performed with the use of the Eco-indicator 99, cumulative energy demand (CED), and Intergovernmental Panel on Climate Change (IPCC) methods. The level of negative effects was determined for the life cycle of a tire and its particular stages: Production, use, and end of life. The negative impact on the atmosphere, soil, and water, as well as on human health, the environment, and natural resources was also investigated. The results show that the most energy-absorbing stage of a car tire life cycle is the use stage. It was found that the most harmful impact involves the depletion of natural resources and emissions into the atmosphere. Recycling car tires reduces their negative environmental impact during all their life cycle stages.

## 1. Introduction

Environmentally focused analyses of car tires involve their description and life cycle assessment in terms of intelligent development, design, the operation and maintenance of machines and technological systems, as well as the decision process from the point of view of science, technology, and the economy [1,2,3]. Knowledge of the analyses results can facilitate the identification of the benefits and drawbacks of engineering objects that can later be used for their comparison and improvement. In the case of production and trade companies, life cycle assessment can be applied to the products themselves and can also address general problems. Their results can be used for the development of action plans, waste management strategies, and design modification to be later compared with other companies [4,5,6]. Companies are more and more frequently using life cycle assessment not only at the stage of product design, improvement, or the formulation of overall development strategies, but also for the object’s entire life cycle management [7,8,9,10,11,12].

In order to develop pro-ecological transport, it is necessary to use data from many different fields. The number of vehicles on the roads is increasing, which has a great impact on the natural environment. To change the situation, joint action needs to be taken by decision makers from more than just one country [13,14].

Legislation imposes waste and product management requirements and deposit fees that producers and importers need to comply with in order to reach a certain car tire recovery level. If they fail to comply with these rules, they are charged with penalty fees [15,16,17].

In view of the above, the ability of the environment to meet the demand that technological systems place on natural resources is rapidly decreasing. This is the reason why the problems relating to the assessment of the environmental impact of car tires need to be addressed [18,19]. An analysis of many papers that deal with the life cycle environmental impact of products such as car tires indicates that it is necessary to provide identification of some sets of impacts and their hierarchy [20,21,22,23,24,25,26]. At the same time, it should be remembered what costs have already been incurred and what costs are likely to be borne in the future. There is also a dependence between the object’s environmental impact and its structural solutions, e.g., a traditional car tire and a tire made of natural rubber [27,28,29,30].

A few studies of the car tire life cycle have already been conducted, yet they were ordered by the manufacturers themselves, which can put the provided results in question. [31,32,33,34]. A respective report was presented by Continental for a 175/70/R13 tire which shows that the highest negative impact occurs at the stage of the tire’s use. However, they do not provide a precise method for calculating its environmental impact. [32]. Another report prepared by Hankook provides general data on greenhouse gas (GHG) emissions and energy consumption during the production process for particular production companies of Hankook, although unfortunately there is no reference to the tire itself [35]. Data on GHG emissions were also presented by Bridgestone [36]. The Evonik company provided the results of a comparative analysis for tires (the tire dimensions were not given), both the traditional ones and those manufactured according to Silica technology with the use of the CML (in Dutch: Centrum voor Milieuwetenschappen, in English: Institute of Environmental Sciences of the University of Leiden) method. It has been proven that the new technology discussed herein contributes to a significant reduction in environmental loads [34]. There are also analyses regarding the carbon footprint of a tire and a retreaded tire which indicate that the environmental impact of the latter is lower [33]. Bras and Cobert performed an LCA analysis for a P205/45R17 tire of Michelin by means of Eco-indicator 99 and the Environmental Development of Industrial Products (EDIP) method, which indicates the tire fuel consumption stage as having the highest environmental impact [31]. Korinek and Koci provide an analysis of a car tire life cycle with the use of GaBi software and the CML 2001 method [25]. Other studies mainly address particular stages of the car tire life cycle, e.g., production [36], but most frequently the end-of-life stage thereof [20,24,37,38,39,40,41]. A few papers present the results of analyses for tires manufactured from Purpose Guayule natural rubber [28,29]. One paper [28] shows that a tire made of a different material is characterized by 6%–30% lower emissions than a traditional tire.

Research on a tire’s end-of-life stage shows, among other things, that the reuse of car tire fibers causes lower environmental load than its combustion with energy recovery [38]. The analyses carried out by Ortiz-Rodriguez et al. by means of the CML 2001 method, on the basis of Columbian experiences, show that the combustion of whole tires in cement kilns is characterized by the lowest environmental impact involved in the recovery of resources [24]. An overall assessment of the end-of-life management of tires is provided by Clauzade et al. [41]. The information they collected on car tire management in France, along with both LCA analysis and the sensitivity of the results thereof, allowed them to state that all methods for car tire recycling/recovery provide environmental benefits [41].

This study provides a comprehensive approach to the assessment of the environmental impact of a P205/55/R16 car tire for three areas: Human health, ecosystem quality, and natural resources. Additionally, the impact on the atmosphere, water, and soil were considered. Energy consumption and CO_2_ emissions were investigated as well. The assessment is based on the Eco-indicator 99 and cumulative energy demand (CED) and Intergovernmental Panel on Climate Change (IPCC) methods.

The main goal of the study was to develop an environmentally focused method for the analysis of a car tire’s life cycle according to the life cycle management (LCM) rules. The research problem was formulated in the form of a question: To what extent and in which areas and stages do car tires contribute to the degradation of the natural environment degradation, to the deterioration of human health, and to the depletion of natural resources during the entire lifetime? In order to solve this problem, a methodology for the assessment and analysis of a car tire life cycle was proposed based on the LCA method. The results are presented in four sections: Introduction, research methodology, results and discussion, and summary with conclusions.

## 2. Materials and Methods

### 2.1. Research Object

The research object is a passenger car tire of P205/55/R16 size (width/profile/diameter). Synthetic (24.17%) and natural (18.21%) rubber constitutes the highest share in the car tire mass, followed by carbon black (19%), coated steel wires (11.4%), precipitated silica (9.65), oil (6.12%), textiles (4.7%), zinc oxide (1.58%), sulfur (1.28%), stearic acid (0.96%), and recycled rubber (0.5%), whose overall weight is about 10 kg (manufacturer’s data).

On average, 6 MJ of electrical energy, 45 L of water, and 0.02 kg of dissolvent are needed to manufacture one tire. The process yields approximately 0.5 kg of waste (manufacturer’s data).

In order to travel 50,000 km, a car that burns 6 L of fuel per 100 km needs approximately 3000 L of fuel. Thus, one tire uses 750 L (manufacturer’s data).

After the end of the use stage, car tires can be utilized in many ways. In European Union countries, they are usually burnt with energy recovery (40%) or subjected to material recycling involving shredding (38%) (data from a tire recycling company). Tires are most often burnt in cement kilns. The end of life for this form of car tire needs 0.06 kg of fuel and 0.06 MJ of electricity per tire. In the case of material recycling for tire shredding, it is necessary to supply 7.4 MJ of electrical energy, 1.5 kg of water, and 0.0001 kg of oils to achieve particles with a size close to 16 × 16 mm. The comminution of a tire to obtain particles of a size below 0.7 mm requires an additional energy input of 5.13 MJ. The processes of pyrolysis use electrical energy of 2.68 to 3.46 kWh/per tire (pyrolysis for combined heat and power (CHP)) (data from a tire recycling company).

### 2.2. Research Methodology

The assessment of environmental impacts was provided for the full life cycle of a car tire. LCA was used to examine the product’s life cycle, starting with production, then the use stage, and ending with end-of-life management. The data used for analysis came directly from the tire manufacturer. The analysis was performed with the use of three methods: Eco-indicator 99, CED, and IPCC. The potential levels of all environmental impacts were analyzed with a special focus on three damage categories: Human health, ecosystem quality, and resources.

#### 2.2.1. Goal and Scope of the Study

The first step of the LCA approach is to determine the research goal and scope by providing a description and analysis of the product life cycle. The next step of the analysis includes inventory analysis, environmental impact evaluation, and the interpretation of results. The LCA is a perfect tool for the assessment of the product’s overall impact on the environment “from the cradle to the grave”. The research involved developing an environmentally focused method for the analysis of a car tire’s life cycle, according to the LCM rules, to discover which areas and stages have the largest potential impact on the degradation of the natural environment, the deterioration of human health, and the depletion of natural resources. Moreover, a detailed objective of the study was to identify the areas with the highest potential harmful impact to the environment. Considering the major objective as well as the more detailed objectives, it was necessary to carry out an analysis of the quality of data and the uncertainty of results. The calculations were made on the basis of the Ecoinvent database library, which was implemented in Sima Pro 8.4.1 (Pré Consultants B.V., Amersfoort, The Netherlands).

The analysis was supposed to describe the flow of materials and energy at different stages of a car tire’s life cycle and provide a quantification and assessment of the emissions and by-products that can have an impact on the natural environment. It was supposed to identify the environmental impact of the tire-use stage as a point of reference to be used for impact reduction. The research goals were to assess the quantitative impact of a car tire’s end-of-life recycling on the natural environment and to develop a normalized assessment method for rubber products. The scope of the analysis included the following stages: Production, use, and recycling. The stages of storing, distribution, sale, and technical tests were not analyzed due to there being too big discrepancies in the data obtained for these phases.

Three assessment methods were used including Eco-indicator 99, CED, and IPPC to determine the environmental loads in different impact areas/aspects. The Eco-indicator 99 method offers 11 impact categories with a wide spectrum of analysis areas, including: Land conversion, acidification and eutrophication, ecotoxicity, carcinogenic substances, climate change, ionizing radiation, ozone layer depletion, respiratory effects caused by inorganic substances, respiratory effects caused by organic substances, fossil fuels, and minerals. Additionally, it is possible to group particular impact categories into specific areas, such as human health, ecosystem quality, natural resources, and emissions to water, soil, and atmosphere, which allows the most affected areas to be identified. It also provides the possibility of comparing different impact groups and weighing the results by reducing them to an environmental unit: Eco-indicator points. The IPCC method was used for the quantitative assessment of CO_2_ emissions to indicate which stage of a car tire’s life cycle causes the highest greenhouse gas emissions (CO_2_), the reduction of which is one of the key assumptions of the European Union countries. The CED method was used to identify the share of a car tire’s life stages in the demand for energy from different sources, including non-renewable energy. In this way, a comprehensive analysis of a car tire’s life cycle, including important areas of sustainable development, such as CO_2_ emissions (IPCC method), energy consumption (CED method), human health, ecosystem quality, and resources (Eco-indicator 99 method) has been provided.

**Figure 1:**
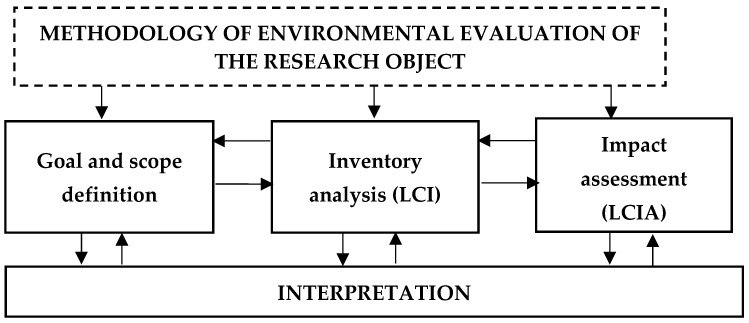
Life cycle assessment (LCA) structure.

**Figure 2:**
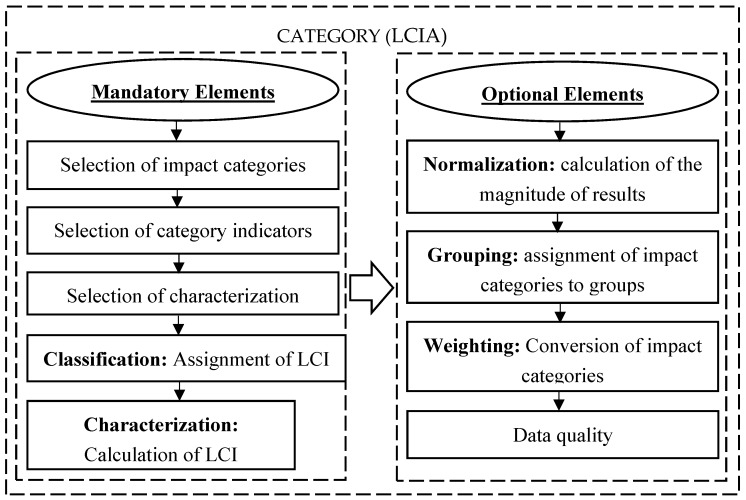
Structure of the life cycle impact assessment (LCIA) phase and relations between its elements.

The basic steps of the LCA analysis include Figure 1: (1) the definition of the research goal and scope, (2) the identification and quantification of loads introduced into the environment, i.e., used materials and energy, as well as emissions and waste, (3) the assessment of the potential load impacts according to the life cycle impact assessment (LCIA) Figure 2 and (4) the estimation of available options to reduce the loads [42,43].

#### 2.2.2. Function and Functional Unit

The data concerning the life cycle of a car tire, which is the research object of this study, can be converted into the accepted function defined as a distance (50,000 km) traveled with a normal style of driving in the 5-year period of the tire-use stage. The selection of a given distance to be a functional unit was based on data on the average tire mileages in Europe [34,35]. One car tire was accepted to be a functional unit.

#### 2.2.3. System Boundary

**Figure 3:**
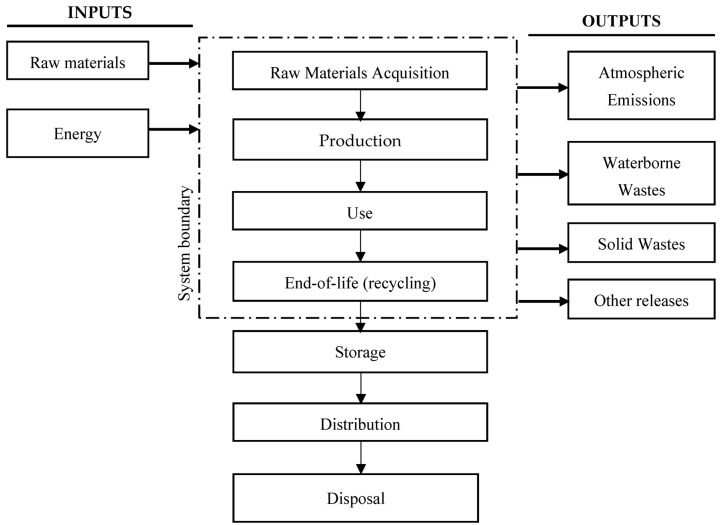
Scope of the life cycle assessment.

The analysis covers almost the entire life cycle of the car tire considered herein, including the stage of raw materials acquisition, production, use, and end-of-life management in the recycling form Figure 3. The stages of storage, distribution, sale, and technical tests were not considered. The time includes the 5-year life cycle of a car tire’s existence.

#### 2.2.4. Analysis of the Input and Output Sets

Special sheets were prepared for data storing. Each sheet was assigned to a single process with a division into the process entries, its implementation, and exits. The entries included the base and auxiliary materials and water; the implementation process included its duration time and consumption of media; and the exits included the main product, waste, and emissions. The data from the sheets was referred to one product. The sheets were being systematically completed along with the progress of the analysis and the obtainment of new information, starting with the data source and proceeding with the place and time of its recording, quality, and finally, validation methods.

The data was acquired in the years 2015–2016. Information concerning the key processes was obtained directly from the manufacturer and from the Ecoinvent database, version 3.2. The data on the materials and media used in the tires are shown in Table 1. Considering the confidentiality of the research results presented herein as well as company trade secrets, the values presented in Table 1 have been changed by a coefficient ranging from 0.8 to 1.2.

#### 2.2.5. Data Allocation

The allocation procedure is set out in the ISO 14044:2006 norm [44] and is of special importance for the analysis of multifunctional processes. Information provided by the manufacturer allowed for the precise quantities of materials and energy used in the life cycle of a car tire to be determined; hence, the allocation procedure is not required.

#### 2.2.6. Data Validation

The data was validated after being assigned to individual processes. Validation involved bilateral energy and mass balancing. Models were built and completed with data. The number of entries balanced the number of exits. Thanks to this, there was data aggregation as well as a conversion of said data into a functional unit and reference streams.

#### 2.2.7. Data Aggregation

Matrixes of entries and exits were obtained for each process through the summation of environmental interventions of the same type (entries of materials, energy, waste, emissions etc.), which were referred to reference streams. In this way, inventory tables were provided. The next step was to adjust them to a format used in the SimaPro program. Thanks to this, it was possible to introduce the data to a computing program and to go on to the next step of the analysis.

#### 2.2.8. Eco-Indicator 99 Method

The Eco-indicator 99 method belongs to the methods used for modeling environmental impact at the level of an environmental mechanism’s final points. The characterizing process is performed for 11 impact categories within the three largest groups which are referred to as impact areas or damage categories. The impact areas include human health, ecosystem quality, and resources [45,46]. The first damage category condenses respiratory and carcinogenic effects, effects on climate change, ozone layer depletion, and ionizing radiation into one value expressed in DALY (disability adjusted life-years). The damage to ecosystem quality is expressed in terms of the percentage of species that have disappeared in a certain area due to the environmental load (percentage of vascular plant species km^2^yr). Ecotoxicity covers the percentage of all species present in the environment living under toxic stress (PAF: Potentially affected fraction). Regarding acidification/eutrophication, the damage to a specific target species (vascular plants) in natural areas is modeled (PDF: Potentially disappeared fraction). The damage category covering resource extraction gives a value expressed in MJ surplus energy to indicate the quality of the remaining mineral and fossil resources Table 2 [47].

#### 2.2.9. CED Method

The CED (cumulative energy demand) method allows one to define the cumulated energy demand. Indicators of the method considered herein are divided into seven impact categories: Two categories of non-renewable resources (nuclear energy, primary forest, and fossil fuels) and five categories of renewable resources (solar energy, wind energy, biomass, water, and geothermal energy) [42,43].

#### 2.2.10. IPCC Method

The IPCC method (Intergovernmental Panel on Climate Change, global warming potential) makes it possible to perform a quantitative assessment of the impact of individual greenhouse gases (GHG) on the greenhouse effect in relation to CO_2_. The timeline within this method is assumed for 20, 100, or 500 years. The overall assessment index of the greenhouse effect impact related to CO_2_ is 1 [48,49]. The global warming potential (GWP) index proposed by the IPCC is a problem-oriented indicator because it merely quantifies GHG emissions (in kilograms of CO_2_ equivalents), not the subsequent climate change related damage (in DALYs) [47].

#### 2.2.11. Characterizing

The characterizing process was carried out for 11 categories of impact, which are placed into three larger groups defined as impact areas and damage categories. The following impact areas can be distinguished: Human health, ecosystem quality, and natural resources [45,46].

#### 2.2.12. Grouping and Weighing

Grouping and weighing was the third step of the car tire life cycle environmental impact analysis. The overall impact of a car tire’s life cycle was taken into consideration. Additionally, the impact on the atmosphere, water, and soil were distinguished, as well as three impact areas: Human health, ecosystem quality, and natural resources depletion.

This step made it possible to provide environmental coefficients. Expressed in Pt (Eco-indicator points), they are aggregated units that make it possible to compare the results of the eco-analyses. One thousand Eco-indicator points is equal to the impact that is exerted by an average European on the natural environment in the span of one year [45]. The cut-off level was equal to 0.05%. The analysis covered 11 impact categories and three impact areas.

#### 2.2.13. Data Quality Analysis

The point of reference used in the assessment of the uncertainty of LCA results is the environmental assessment of the life cycle treated as a specific measurement method based on a given methodology. Its application leads to the ‘measurement’ of two basic elements: The environmental aspects that occur in the product’s life cycle as well as the subsequent potential environmental impacts. The term ‘uncertainty’ has a number of interpretations, including those that exclude related concepts such as variability and sensitivity. Therefore, it is difficult, or even impossible, to provide a generally applicable and accepted definition of uncertainty. Therefore, in practice, it is necessary to define points of reference that are assumed to represent the truth. Typical examples of such points of reference would be measurements. If we trust the measurement method and the protocol, we believe that a measurement represents a kind of truth in a specific point of space and time, and the difference between the estimation result and an equivalent measured value is a definition of the uncertainty index [50].

According to one international dictionary of metrological terms, “uncertainty is a parameter connected with the result of measurement characterizing the scatter of values, which can be attributed to the measured quantity in a justified manner. Uncertainty characterizes the scatter of values (the interval width), within which the measured quantity value can be placed with a satisfying probability [51]”. For LCA, it means that uncertainty is included in the results of “measurements” obtained for different research levels, i.e., from the analysis of the set of entries and exits relating to the environmental aspects (LCI results) and from the stage of the assessment of life cycle impact on the natural environment (LCIA results).

It is noteworthy that the term “sensitivity” is used in various and inconsistent ways in the literature, and no agreement on its exact definition exists. Two main uses can be distinguished: (1) For some authors, sensitivity includes the effect of uncertainty and thus considers the range of variation of input parameters as a function of their uncertainty (which hence needs to be known), varying them all at the same time. This is also called global sensitivity analysis, and is essentially what this chapter refers to as uncertainty analysis. (2) Others define sensitivity solely as the effect of a certain input change on the output, applying a predefined variation without considering the uncertainty. This is analyzed by varying one parameter at a time, and is also called local sensitivity analysis.

Triangular distributions based on two parameters determining the minimum and the maximum values were defined for the database accepted in the study. The results of data quality assessment with the use of origin matrixes are presented as data quality indicators (DQI). If the maximum DQI value was 5, and the minimum was 1, each assessed inventory element could reach one of 21 DQI values Table 3. The highest assessment with the use of an origin matrix indicated the highest data quality. It meant that when one of the inventory data obtained a DQI = 5 value, it could be trusted, but in practice, a slight deviation of this value could be expected. The opposite happened when one of the inventory data reached DQI = 1, which means low quality and high uncertainty [47].

Being in possession of data collected from a company, an inventory table was prepared with areas that make it possible to introduce uncertainty information. Normal distributions were accepted for analysis. Data quality assessment was performed by means of the method of origin matrixes, which includes the following elements: Reliability, completeness, a timeline, geographic range, and technological range. The deviation levels are presented in Table 4.

Knowing the values of deviations, it is possible to determine the minimum and the maximum for each inventory element Table 5. By using triangular distributions and treating the value presented in the inventory table as the most probable and knowing its DQI, the minimum and the maximum could be determined [47]. Knowing the distribution type and the values of respective parameters, the program performs simulations that make a random choice of the values of consumption of water, energy, resources, and emissions.

An analysis of uncertainty was performed simultaneously for all the inventory elements in the system including information on their uncertainty. Thus, 1000 results of LCIA (life cycle impact assessment) were provided. The Sima Pro computing program automatically created a histogram for each analyzed impact category, and from the set of LCIA calculated the basic parameters thereof, such as standard deviation, the mean, median, and variability coefficient results [47].

## 3. Results and Discussion

### 3.1. Total Impact

**Figure 4:**
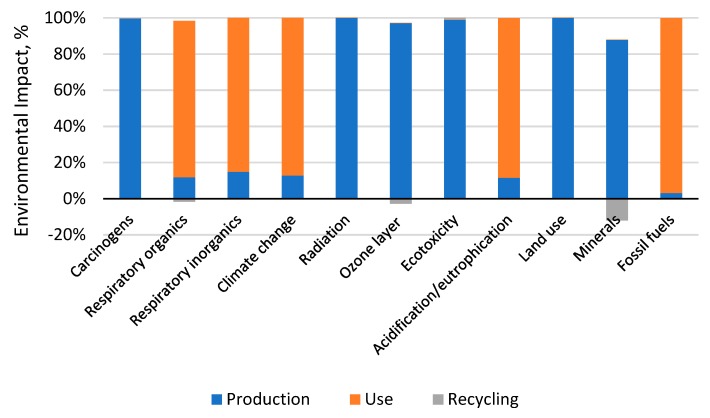
Results of grouping and weighing environmental impacts occurring in the life cycle of a car tire, taking into account the category of impacts.

Discussion of the results is limited to the results of the third stage of the LCA analysis, that is, to grouping and weighing. The results concerning environmental impacts for the 11 impact categories are presented in Figure 4. Out of the 11 impact categories, those for which the sum of environmental impacts was higher than 90% were found to be relevant [52]. The following impact categories were found to be relevant for a car tire’s entire life cycle Table 6: Fossil fuels, respiratory inorganics, and climate change. The same categories turned out to be relevant at the stages of tire use and recycling. Additionally, the category of carcinogenic substances was found to be significant for the production stage.

**Figure 5:**
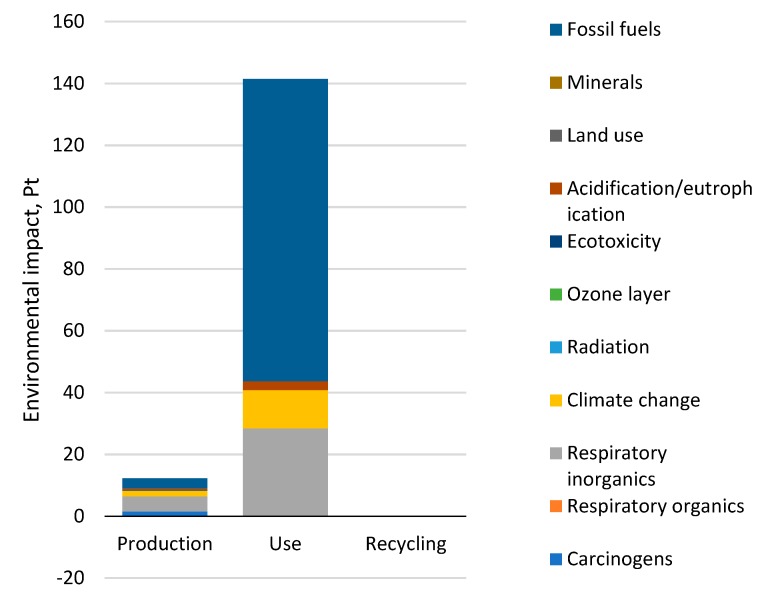
Results of grouping and weighing environmental impacts occurring at the stages of the life cycle of a car tire.

The results show that over 80% of potentially negative impacts occur at the stage of a car tire’s production, including: Carcinogens compounds, radioactive compounds, compounds that increase ozone depletion, ecotoxic compounds, land use, mineral extraction Figure 4—Which is mainly related to the use of materials such as natural and synthetic rubber—steel cords, textile fibers, oils, stearic acids, zinc oxides, copper, as well as energy use in the technological processes involved in the production of these materials to combine them into a single product: A car tire. The values of the remaining categories—meaning respiratory organic, respiratory inorganic, climate changes, acidification/eutrophication, and the extraction of fossil fuels—Are generated in more than 80% of cases during a car tire’s operation stage, which also features the highest summary environmental impact (Figure 4 and Figure 5) and is caused mainly by the consumption of fuel throughout the lifetime. Extension of the car tire life cycle will lead to the introduction of additional emissions and decrease the number of tire changes, which in turn could efficiently reduce the demand for tires and contribute to a reduction in the harmful impacts arising from the production stage and life-end-use management on a global scale.

**Figure 6:**
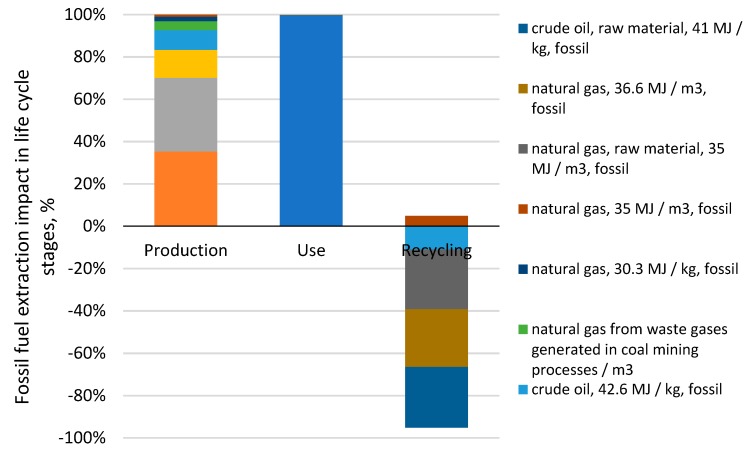
Results of grouping and weighing of environmental impacts for the processes connected with the extraction of fossil fuels involved in a car tire life cycle.

The highest level of negative environmental impacts was found for the category of fossil fuels extraction processes, specifically in the use stage, which came to 97.8 Pt and is caused by fuel consumption at the use stage. Among all the processes analyzed Figure 6, the highest impact on this category was exerted by the process of natural gas extraction, accounting for 100% of the harmful impacts at the use stage and throughout the tire life cycle (contribution: 96.85%). The extraction of natural gas (contribution: 35.17%) and oil (contribution: 34.85%) was found to have a significant impact on the environment. They can be indicated as hot spots in the category of fossil fuel extraction at the use stage (Figure 6). Other relevant processes at the use stage are hard coal extraction (contribution: 13.28%), crude oil, 42.6 MJ/kg, and fossil extraction (contribution: 9.29%).

**Figure 7:**
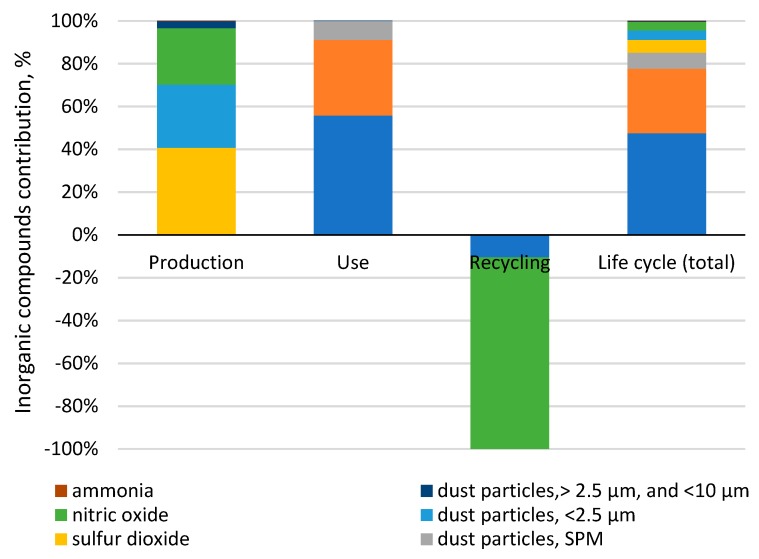
Share of inorganic compounds that cause respiratory system disorders in the impact category “respiratory inorganics”.

The categories of inorganic compounds—which cause respiratory diseases—and compounds that cause climate change have a particularly negative impact on the environment. Among inorganic compounds, the most harmful ones are sulfur oxide emissions (contribution: 55.8%) and nitrogen dioxide (contribution: 35.31%) from fuel combustion during the use stage. As regards the first compound, the value came to 15.8 Pt, and for the second, it came to 10.0 Pt. In this category, four substances are significant: Sulfur oxide, nitrogen dioxide, dust particles SPM (suspended particulate matter), and sulfur dioxide Figure 7.

**Figure 8:**
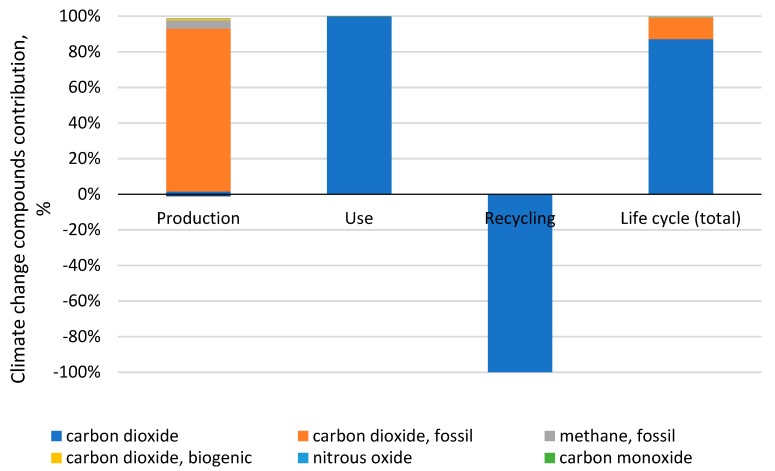
The share of substances in the impact category “climate change”.

It was found that the most potentially harmful environmental impacts involved in the stage of a car tire’s use belonged to the category of compounds that cause climate changes, with a value of 12.41 Pt. Emission levels in this category were shaped mainly by carbon dioxide emissions during the use stage at 12.4 Pt, which were emissions that relate mainly to the use of fuel while driving a car. In the category of climate change, carbon dioxide (almost 99%) was found to be the most harmful. At the production stage, carbon dioxide constituted the greatest share (93.7%) Figure 8.

The results of grouping and weighing environmental impacts for cancerogenic compounds generated during a car tire’s life cycle showed that the highest potentially negative environmental impact was found in the production stage, this coming to 1.5 Pt. Arsenic and cadmium ions were found to have the highest values in this category. For arsenic ions, the value was 1.35 Pt, and for cadmium ions, it was 0.12 Pt. In this impact category, it was found that the biggest share was constituted by arsenic ions (almost 89%) and cadmium ions (about 8%) (Figure S1).

It was found that the highest potentially harmful environmental impact of organic compounds that cause respiratory diseases belonged to the use stage, with a value of 0.0171 Pt. In the case of a traditional car tire, hydrocarbons, which exert the most harmful influence on people and animals, were found to be at the level of 1.71 × 10^−2^ Pt. In turn, at the stage of production, it was found that the highest emissions for the category of impacts belonged to non-metal volatile organic compounds (NMVOC), with a value of 1.71 × 10^−3^ Pt. In this impact category, unspecified hydrocarbons (contribution: Almost 90%) and NMVOC, non-methane volatile organic compounds (contribution: 7.4%) were found to be relevant (Figure S2).

In the radiation category, it was found that the biggest share belonged to radon-222 (contribution: 67.5%) and carbon-14 (32%) for the entire life cycle of a car tire (as well as at particular life cycle stages) (Figure S3). It was found that among substances causing ozone layer depletion, the biggest share belonged to bromotrifluoromethane, halon 1301, and bromochlorodifluoromethane, halon 1211 (Figure S4). Ecotoxicity in a car tire’s life cycle was mainly caused by seven substances: Zinc, nickel, copper ions, nickel ions, lead, chrome, and mercury, which are generated at the production stage (Figure S5). For the eutrophication/acidification category, three compounds were found to be relevant: Nitrogen dioxide (60.3%), sulfur oxide (28.3%), both of which are generated during the use stage, and nitric oxide (7.8%), which mainly occurs at the production stage (Figure S6). In the minerals category, nickel (contribution: 49%), copper (32.7%), and molybdenum (11%) were found to be relevant (Figure S7). Worthy of note is that the recycling stage reduces the negative impact of iron extraction by almost 90%.

The results unequivocally show that the highest potentially harmful impact on the natural environment is caused by fuel consumption at the stage of a car tire’s use. Similar results are presented in papers [31,32]. Bras and Cobert [31] show that the stage of fuel consumption (consistent with the use stage) potentially causes the highest impacts in the category of fossil fuel consumption and inorganic compounds, which is consistent with the results presented in this study. However, the values of environmental impacts expressed in Pt, which in paper [31] were lower than the impact categories presented above, make up the difference. These differences might be caused by the fuel consumption level (lower in paper [31]) during a car tire life cycle, which depends on the type of fuel and the geographic location of the resources and energy. In his report, Continental [32] also indicates the car tire use stage as being the most harmful to the environment as well as the fossil fuel extraction process, including natural gas and oil extraction, although the slightly different impact categories should be taken into account here.

This study has proven that it is the production process (in terms of materials and electric energy consumption) that has the most destructive impact on the natural environment in terms of the emission of inorganic substances that cause respiratory diseases, which is consistent with the results published in [36].

In the light of the above, it must be said that in order to improve the negative impact of car tires on the environment, it is necessary to find effective methods to reduce the environmental impact of the use stage, thus limiting the additional consumption of fuel brought on by the use of tires. Hence, it is necessary to reduce the resistance of rolling. Changes to be introduced at the use stage should include decreasing energy consumption as well as replacing raw materials and minerals with recyclables. The application of recycling makes it possible to reduce environmental impacts, particularly with regard to mineral extraction Figure 4. The development of an efficient method for car tire end-life processing is a challenge from both the technological and legislative points of view. It also requires the development of detailed methods and the provision of directives on waste management. Increasing the share of energy and material recovery methods, preferably the implementation of the procedures of the so called ‘closed circuit’ economy, seems to be of particular importance. This study uses the data of the European energy mix which, according to the estimates of Ecoinvent Centre, has the lowest values of environmental emissions per 1 kWh of energy [53]. The use of energy mix from the USA or China could cause significant changes in the share of particular life cycle stages in the context of environmental impacts. The use of a database for China would provide the highest values of environmental impacts, as, according to the Ecoinvent Centre, its environmental emission index values are the highest per 1 kWh of energy [53].

### 3.2. Impact on the Atmospheric, Aquatic, and Soil Environment

**Figure 9:**
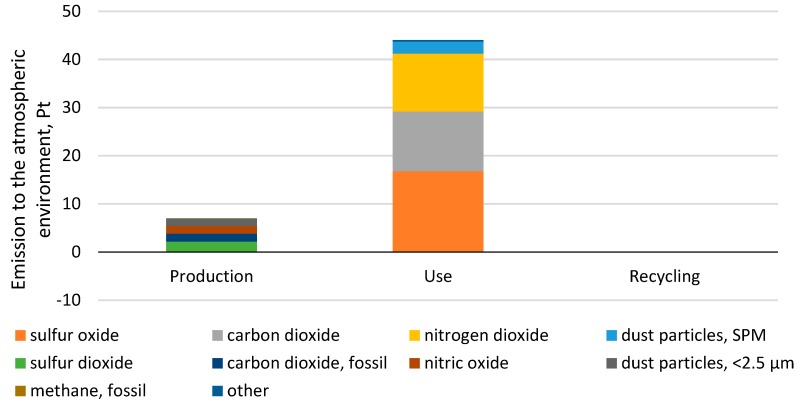
Results of grouping and weighing environmental impacts in relation to emissions into the atmospheric environment throughout the life cycle of a car tire.

The most harmful substances affecting the atmosphere are emitted during the use of car tires, with this being 43.63 Pt Figure 9). The highest share in the generation of substances such as sulfur oxide, carbon dioxide, nitrogen dioxide, and SPM dust can be attributed to a car tire’s use stage. The largest negative impact is reported for emissions of sulfur oxide, which is equal to 16.75 Pt during the use stage. In addition, emissions of carbon dioxide and nitrogen dioxide adversely affect the atmosphere. A significant share of the toxic emissions produced during the car tire use stage results mainly from fuel consumption. A significant amount of fuel is combusted at the stage of a car tire’s use, and the exhaust emissions are released into the atmosphere. The results indicate that these are the main substances that cause acid rain. It is the stage of car tire production that contributes the most to emissions of sulfur dioxide, CO_2_, nitric oxide, and dust particles below 2.5 μm, which are generated from electric energy consumption during the production process. Recycling makes it possible to reduce the harmful impacts of car tires on the atmosphere throughout their lifetime by 1.03 × 10^−2^ Pt. Each reduction in fuel consumption at the use stage will cut emissions into the atmosphere. According to the Evonik report, a fuel consumption reduction of 2% will decrease CO_2_ emissions by up to 40% [34]. In their study, Korinek and Koci [25] also show that during the lifetime of a car tire, the emissions of CO_2_ and sulfur dioxide are the most abundant.

The largest amount of pollution arising during a car tire’s production gets into the water, the total impact being equal to 1.53 Pt Table 7. The production stage was found to have the highest impact of arsenic. Arsenic ions are particularly harmful to the aquatic environment, causing harmful impacts that come to a value of 1.35 Pt. Arsenic is one of the microelements, but its excess can result in severe poisoning. All arsenic compounds are characterized by protoplasmic properties (destroying cell walls) and are cancerogenic (contributing to cancers of the skin, lungs, kidneys, liver, and bladder).

The production stage has the highest share in the environmental impacts on the soil (4.69 × 10^−3^ Pt). The elements that appear at the production stage and cause the largest environment damage are cadmium, zinc, and chromium (Table 8).

**Figure 10:**
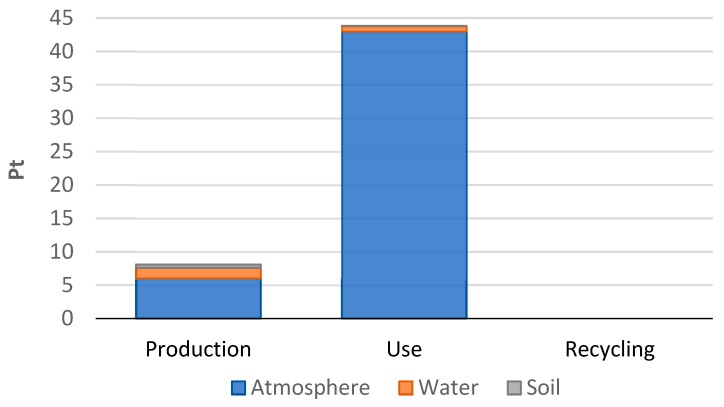
Results of grouping and weighing environmental impacts in relation to emissions to the atmospheric, aquatic, and soil environments during the stages of a car tire’s life cycle.

A car tire’s use stage has the largest summary environmental impact in the three areas: Atmosphere, water, and soil (44 Pt). It was found that emissions make up the highest share of this stage. Emissions, for the most part, are caused by the consumption of fuel that is combusted in the vehicle drive unit, as a result of which substances such as carbon dioxide, nitrogen dioxide, and sulfur oxide are released into the atmosphere Figure 9. In terms of the entire car tire life cycle, it was the share of atmospheric emissions that was found to be particularly high (96%) as compared to other emission types. Small quantities of harmful compounds also get into the aquatic and soil environment (4%) Figure 10.

### 3.3. Impact on Human Health, Ecosystem Quality, and Natural Resources

As regards the impacts on human health, the highest values were found for the use stage, with a value of 40.79 Pt. Fuel consumption involves the generation of three major substances Table 9. The first of them is sulfur oxide, which is generated during use of tires, and has a harmful effect on human health. This value came to 15.8 Pt. The second substance is carbon dioxide, whose impact was measured at 12.4 Pt. The third substance is nitrogen dioxide, whose impact is at the level of 10.0 Pt. The production stage, in turn, has a share of substances harmful to human health, as it generates sulfur dioxide, dust particles <2.5 μm, carbon dioxide from fuel mines, arsenic ions, and nitric oxide.

**Figure 11:**
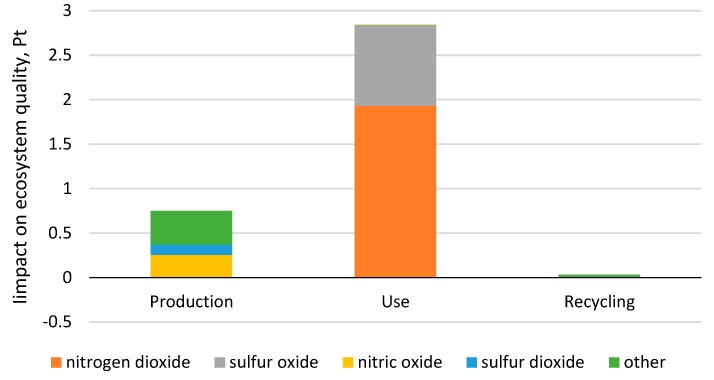
Results of grouping and weighing environmental impacts for compounds affecting the ecosystem quality throughout the stages of the life cycle of a car tire.

The biggest environmental threat in the category of “ecosystem quality” involves the stage of a car tire’s use, the negative impact of which comes to 2.84 Pt Figure 11. Emissions of nitrogen dioxide and sulfur oxide have a more harmful impact on the ecosystem quality than all the substances analyzed for this impact area, and it is the car tire use stage (more specifically, fuel consumption) that is responsible for this harmful impact. The value of nitrogen dioxide’s impact is 1.93 Pt., whereas that of sulfur oxide is equal to 0.91 Pt. The production stage, in turn, has the highest share of nitric oxide generation and sulfur dioxide generation in the area of environmental impact.

Most harmful to the environment in the fossil fuel area of a car tire’s life cycle is the depletion of natural gas and oil. A study of Chinese tires [54] has provided similar conclusions, although the analyses take advantage of a different impact assessment method (CML). The use stage was found to have the biggest impact in this area, with a value of 97.83 Pt Table 10. This stage contributes to 98% of natural gas depletion, which results mainly from the fuel consumption involved in this stage. Each change in fuel type will cause changes in the area of environmental impacts on natural resource depletion. If car fuel was replaced by electrical energy, it would be possible to reduce the impact of the car tire life cycle (especially its use stage), assuming that analyses for the energy mix are carried out in which renewable energy sources would prevail.

The depletion of crude oil deposits is almost 100% due to car tire production processes, which is mainly related to the use of rubber in tire production. The impact of this stage in terms of natural gas consumption is also significant, which is caused by energy consumption during the production processes. Recycling processes have only a slight impact on natural resource depletion through energy recovery.

**Figure 12:**
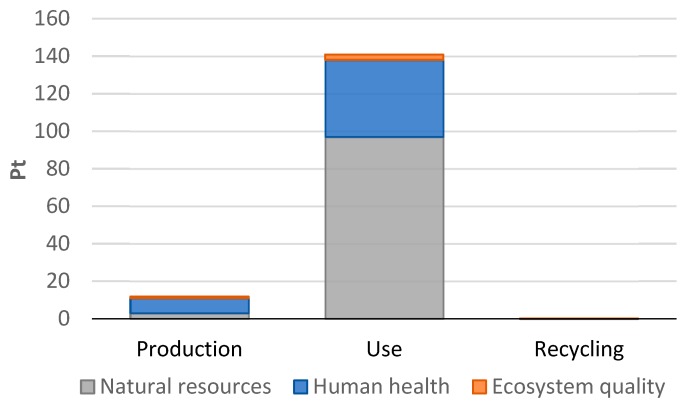
Results of grouping and weighing environmental consequences for compounds affecting human health, ecosystem quality, and raw material resources throughout the stages of the life cycle of a car tire.

The most harmful stage of a car tire’s life cycle is the use stage for each of the areas being considered Figure 12.

From the perspective of the entire life cycle of a tire, these are the fossil fuel extraction processes that have the most negative impact on the ecosystem quality (66%). They have also a detrimental effect on human health (32%). This confirms the correctness of the assumption that passenger car engine fuel should be switched to a more pro-ecological one and that fuel consumption should be reduced. As in this study, Continental found in his report that the extraction of resources was highest in the use stage [32]. In study [54], similar conclusions were presented, although the analysis was performed within the geographical borders of China and the energy mix of this country was used.

### 3.4. Energy Consumption Assessment

**Figure 13:**
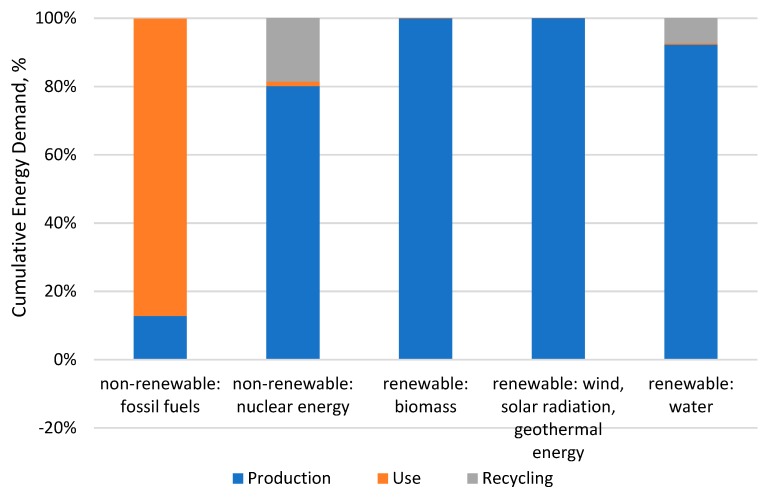
Percentage share of life cycle stages of a car tire in cumulative energy demand from different sources.

Table 6 presents the results of energy consumption in each stage of a car tire’s life cycle with the use of the CED method. It was found that the largest influence on the overall energy demand (28,544 MJ) belonged to the use stage (fuel consumption) Table 11. The values obtained for total energy consumption were lower than those presented in [32], which may be caused by the type of tire used in the analysis (in [32], a tire of R13 size) and the method for assessing energy consumption (including national data on energy mix). Comparing the energy consumption results, the values obtained are about 20 times lower than in the Evonik report [34], which was mostly the result of assuming that the life cycle is three times longer (150,000 km) than the one assumed in this study. It was found that fossil fuel energy made up the highest energy consumption during a car tire’s life cycle, whereas the use stage contributed to more than 80% of this consumption Figure 13. As much as 80% of energy from other sources is consumed at the production stage. Recycling processes make an insignificant contribution to the reduction of fossil fuel energy consumption (by about 0.10%). Despite energy consumption being reduced in the area of fossil fuel energy production, the recycling processes contribute to an increase in energy consumption from other sources: Nuclear energy (by about 20%) and hydro energy (by about 10%) Figure 13.

It needs to be emphasized that the energy consumption results were obtained for the European energy mix, of which renewable energy sources still make up only a small share. Switching the energy mix from the European one to a different one, e.g., to that of America, China, or for instance to that of particular European countries such as Poland, France, Germany, or Spain would involve significant changes in energy consumption from particular sources. According to the Ecoinvent 3.2 database, in Poland, 1 kWh of electric energy is produced mainly from hard coal (0.56 kWh) and brown coal (0.37 kWh), which accounts for nearly 91% of all energy sources used in the Polish energy mix. The remaining components of the mix include energy produced from industrial gases, natural gas, hydro energy, and wind energy, as well as others [55]. When it comes to analyzing the French energy mix, it turns out that in this country, most energy comes from nuclear power plants, nearly 80% of it [53].

If it were assumed that the fuel used differed from the one analyzed herein (thus, a different kind of vehicle drive), e.g., gasoline or electric energy, completely different results would be obtained with regard to the consumption of energy from different sources for the stages of a car tire’s use. The same applies to the assumption regarding the amount of fuel to be burned.

First of all, a reduction in energy consumption during a car tire’s lifetime should involve increasing the efficiency of fossil fuel energy production as well as decreasing the consumption of energy involved in the processes of a car tire’s production and recycling.

### 3.5. Greenhouse Gas Emissions

The results of greenhouse emissions analysis for each stage of a car tire’s material life, made with the use of the IPCC method, are presented in Table 12. The highest level of GHG emissions into the atmosphere was 2265 kg CO_2_ eq. A large impact on the environment in this life-cycle stage is caused by the combustion of a large amount of fossil fuels by a passenger car to set the wheels and tires in motion. The values of total greenhouse emissions provided were lower than those presented in report [34], which results, as in the case of energy consumption, from the application of a different tire type, a different research method. Furthermore, what is also significant in the Evonik report [34] is that the tire mileage was assumed to be 150,000 km—that is, a three times higher value than the one accepted in this study. In turn, in report [32] and in study [31], the emissions were lower. Differences may be the result mainly of inventory data, which in study [31] referred to the United States, while in report [32], lower fuel consumption (at the level of 5.2% of the vehicle’s total consumption) was assumed for the use stage.

### 3.6. Uncertainty Analysis

**Figure 14:**
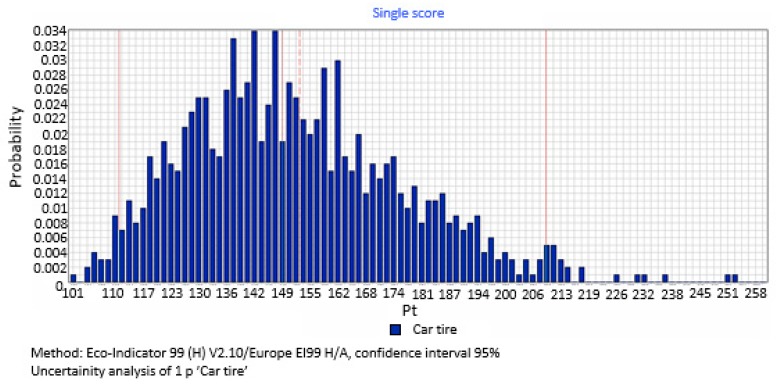
Results of uncertainty analysis for the entire life cycle of a car tire.

Figure 14 shows the results of uncertainty analysis for the process of a car tire’s production, use, and recycling. The mean value is 153 Pt. The remaining parameters of distribution are the following: Median = 149 kPt, standard deviation = 37.2 Pt, and variability coefficient = 24.3%. Diversification (dispersion) of the final results is relatively small, and it can be stated that the value of the median offers a good reflection of the average level of the phenomenon studied herein (the environmental impact of a car tire’s entire life cycle).

According to the example presented, the final result of uncertainty analysis carried out as part of full LCA research should be summarized as follows: Based on the data acquired, it can be concluded that the result of the eco-index for the production, use, and recycling of a car tire during the entire life cycle is 153 Pt, with a standard deviation at the level of 37.2 Pt, which means that there is a 95.5% probability (±2 σ) that the eco-index result will range from 111 to 209 Pt for the car tire. Such a conclusion is consistent with the definition of uncertainty quoted in Section 2.2.13, according to which the scatter of values (the interval width), within which the value of a measured quantity can be placed with a satisfying effect, is characterized by uncertainty [47].

## 4. Conclusions

The most important task to be accomplished in order to achieve the main goal of the study—To identify the areas with the most harmful environmental impact—was made possible through an ecological energy analysis of a car tire’s life cycle. The use stage generates the largest amount of environmental impacts (total: 141 Pt). The following impact categories are shown for a car tire’s entire life cycle: Fossil fuels (contribution: 65.67%), respiratory inorganics (contribution: 21.71%), and climate change (contribution: 9.25%). The process of natural gas extraction was found to have the highest environmental impact on the category of fossil fuels exhaustion, accounting for 100% of the harmful impacts at the use stage and throughout a tire’s life cycle (contribution: 96.85%). The extraction of natural gas (contribution: 35.17%) and oil (contribution: 34.85%) was found to have a significant impact on the environment. For the respiratory inorganics category, four substances were found to be significant: Sulfur oxide, nitrogen dioxide, dust particles SPM, and sulfur dioxide Figure 7. For the category of climate change, carbon dioxide (almost 99%) was found to be significant. In a car tire’s entire life cycle, arsenic ions (almost 89%) and cadmium ions (about 8%) made up the biggest contributions to the cancerogenic compounds. For the respiratory organics category, unspecified hydrocarbons (almost 90%) and NMVOC, non-methane volatile organic compounds (7.4%), were found to be significant. For the radiation category, radon-222 and carbon-14 made up the biggest share in harmful impacts for the entire life cycle of a car tire (and all the stages) at 67.5% and 32%, respectively. Among the substances causing ozone layer depletion, the biggest share belonged to bromotrifluoromethane, halon 1301, and bromochlorodifluoromethane, halon 1211. Ecotoxicity in a car tire’s life cycle was caused mainly by seven substances: Zinc, nickel, copper ions, nickel ions, lead, chromium, and mercury, all of which are generated at the production stage. For the eutrophication/acidification category, three compounds were found to be significant: Nitrogen dioxide (60.3%) and sulfur oxide (28.3%)—which are generated in the use stage—and nitric oxide (7.8%), which occurs mainly in the use stage. For the minerals extraction category, it was the extraction of nickel (contribution: 49%), copper (32.7%), and molybdenum (11%) that had the highest environmental impact. This study has proven that in the context of materials and electric energy consumption, the aspect that is most destructive to the natural environment in terms of the emissions of inorganic substances that cause respiratory diseases is the production process. The use of conventional fossil fuels while operating a passenger car tire has a large negative influence on the natural environment. In order to decrease fuel consumption, tires should be modified by reducing their rolling resistance. However, taking into consideration that fuel consumption mostly depends on vehicle type, changing the fuel type and engine type will cause a change in the car tire’s environmental impacts. Each fuel application reduction will decrease the tire’s environmental impact. Assuming that the electric energy comes from renewable energy sources, changing the drive, i.e., to an electric one, could significantly reduce the environmental impact in the area of emissions into the atmosphere and the depletion of natural resources.

The highest negative impact on human health, ecosystem quality, and resources occurs during the use stage of a car tire’s life cycle. The processes related to the extraction of fossil fuels have the most negative impact on the environment (66%). They also have an adverse effect on human health (32%). Emissions of three substances—sulfur oxide, carbon dioxide, and nitrogen dioxide oxide—are of key significance in the area of impact on human health. Emissions of nitrogen dioxide and sulfur oxide have the highest level of harmful effects that impair the quality of the environment (among all the substances analyzed in this impact area). In turn, the highest level of harmful impacts in the area of natural resources exhaustion proved to be natural gas extraction processes.

From the perspective of a tire’s entire life, the highest level of harmful emissions was reported for the atmospheric environment (96%). The stage of a car tire’s life cycle absorbing the most energy is the use stage. The recycling of car tires reduces their negative impact in all stages of the life cycle to a small degree. In order to decrease the impact of tires, it is necessary to introduce more efficient recycling methods that would make it possible to use the materials and components from car tires as efficiently as possible and decrease energy use to prevent additional environmental loads.

The final result of uncertainty analysis carried out as part of complete LCA research should be summarized as follows. Based on the data acquired, it can be concluded that the result of the eco-index for the production, use, and recycling of a car tire throughout the life cycle is 153 Pt, with a standard deviation at the level of 37.2 Pt, which means that there is a 95.5% probability (±2 σ) that the eco-index for car tires will range from 111 to 209 Pt.

## Figures and Tables

**Table 1:** Life cycle inventory analysis: Car tire.

Total Consumption per 1 Tire Manufactured	
Input	Quantity
**Production stage**	
synthetic rubber [kg]	1.928
natural rubber [kg]	1.456
carbon black [kg]	1.496
precipitated silica [kg]	0.768
sulfur compounds [kg]	0.096
zinc oxide [kg]	0.128
mineral and plant oils [kg]	0.472
stearic acid [kg]	0.08
rubber from recycling [kg]	0.04
steel wires [kg]	0.888
textiles [kg]	0.368
polymer substances-polyurethanes [kg]	0.192
ethyl acetate [kg]	0.024
substance facilitating rubber gluing-butadiene adhesives [kg]	0.024
antiadhesive substances-silicone [kg]	0.2
remaining solvents [kg]	0.016
water [L]	36.112
electricity [MJ]	828.896
others [kg]	0.096
**Use stage**	
furnace oil for power generation [L]	5.12
high-speed diesel for power consumption [L]	0.2716
**Recycling stage**	
used tire [kg]	40
water [L]	36.112
electricity [MJ]	828.896
mineral and plant oils [kg]	0.472

**Table 2:** Dependence between life cycle inventory (LCI) data and damage categories in the Eco-indicator 99 methodology. PAF: Potentially affected fraction, PDF: Potentially disappeared fraction, DALY: disability adjusted life-years.

Life Cycle Inventory (LCI)	Effect	Damage Category
Extraction of minerals and fossil fuels	Surplus energy for future extraction	Damage to mineral and fossil resources (MJ surplus energy)
Land use: Occupation and transformation	Occurrence of vascular plant species (POO)	Damage to ecosystem quality (percentage of vascular plant species km^2^yr)
NO_x_, SO_x_, NH3	Acidification/eutrophication (PDF)	
Pesticides, heavy metals	Ecotoxicity: Toxic stress (PAF)	
CO_2_, Hydrochlorofluorocarbons (HCFC)	Climate change	Damage to human health (DALY)
Hydrochlorofluorocarbons (HCFC)	Ozone layer depletion	
Nuclides	Ionizing radiation	
Suspended particulate matter (SPM), volatile organic compounds (VOCs), NO_x_, SO_x_	Respiratory effects	
Polycyclic aromatic hydrocarbons (PAHs)	Carcinogenics	

**Table 3:** Data quality indicator (DQI) values and deviation levels.

DQI	Deviation [%]	DQI	Deviation [%]	DQI	Deviation [%]	DQI	Deviation [%]
5.0	10	4.0	20	3.0	30	2.0	40
4.8	12	3.8	22	2.8	32	1.8	42
4.6	14	3.6	24	2.6	34	1.6	44
4.4	16	3.4	26	2.4	36	1.4	46
4.2	18	3.2	28	2.2	38	1.2	48
-	-	-	-	-	-	1.0	50

**Table 4:** Data quality criteria for a car tire LCA assessment.

Criterion	Target Level
Reliability (precision)	Verified data, based on measurements
Representativeness	Data from a production company
Age of data	The year 2017
Geographic origin	Poland
Technological representativeness ep	Tire manufacturing technology used in the automotive industry in Poland

**Table 5:** Inventory table of data quality analysis for the car tire process.

Material	Min	Max
**Production Stage**		
synthetic rubber [kg]	2.169	2.651
natural rubber [kg]	1.638	2.002
black carbon [kg]	1.683	2.057
precipitated silica [kg]	0.864	1.056
sulfur compounds [kg]	0.108	0.132
zinc oxide [kg]	0.144	0.176
mineral and plant oils [kg]	0.531	0.649
stearic acid [kg]	0.09	0.11
rubber from recycling [kg]	0.045	0.055
steel wires [kg]	0.999	1.221
textiles [kg]	0.414	0.506
Polymer-polyurethanes [kg]	0.216	0.264
ethyl acetate [kg]	0.027	0.033
substance facilitating rubber gluing-butadiene glues [kg]	0.027	0.033
antiadhesive substance-silicon [kg]	0.225	0.275
remaining solvents [kg]	0.018	0.022
water [L]	40.626	49.654
electric energy [MJ]	932.508	1139.732
others [kg]	0.108	0.132
**Use stage**		
furnace oil for power generation [L]	5.248	7.552
high-speed diesel for power consumption [L]	0.27839	0.3285002
**Recycling stage**		
used tire [kg]	45	55
water [L]	40.626	49.654
electric energy [MJ]	932.508	1139.732
mineral and plant oils [kg]	0.531	0.649

**Table 6:** The share of impact categories in the car tire life cycle (relevant impact categories).

Impact Category	Contribution *, %			
	Life Cycle (Total)	Production	Use	Recycling
Carcinogens	0.981	12.309	0.001	2.232
Respiratory organics	0.013	0.019	0.012	0.190
Respiratory inorganics	21.712	40.431	20.077	12.263
Climate change	9.247	14.754	8.766	6.353
Radiation	0.002	0.022	0.000	0.000
Ozone layer	0.000	0.003	0.000	0.006
Ecotoxicity	0.149	1.842	0.000	1.237
Acidification/eutrophication	2.089	3.024	2.008	1.917
Land use	0.126	1.573	0.000	0.000
Minerals	0.007	0.100	0.000	0.939
Fossil fuels	65.675	25.922	69.137	77.337

* Impact categories that were found to be relevant for the entire car tire life cycle and for particular life cycle stages are marked in orange.

**Table 7:** Results of grouping and weighting environmental impacts in relation to emissions into the aquatic environment over the life cycle of a car tire.

Substance	Production, Pt	Use, Pt	Recycling, Pt
arsenic, ions	1.35 × 10^0^	7.79 × 10^−4^	−4.62 × 10^−3^
cadmium, ions	1.17 × 10^−1^	9.46 × 10^−5^	−1.37 × 10^−4^
copper, ions	2.85 × 10^−2^	1.29 × 10^−4^	−7.52 × 10^−5^
nickel, ions	2.79 × 10^−2^	2.51 × 10^−5^	−7.45 × 10^−5^
hexavalent chromium	4.47 × 10^−3^	2.59 × 10^−5^	-
zinc, ions	2.62 × 10^−3^	1.28 × 10^−5^	−1.70 × 10^−5^
mercury	2.50 × 10^−4^	4.39 × 10^−6^	2.54 × 10^−8^
lead	1.64 × 10^−4^	1.59 × 10^−6^	−3.60 × 10^−6^
caesium-137	2.75 × 10^−6^	1.21 × 10^−10^	-

**Table 8:** Results of grouping and weighing environmental impacts in relation to emissions into the soil environment over the life cycle of a car tire.

Substance	Production, Pt	Use, Pt	Recycling, Pt
cadmium	2.99 × 10^−3^	1.35 × 10^−8^	-
zinc	1.03 × 10^−3^	1.47 × 10^−7^	-
chromium	3.78 × 10^−4^	6.91 × 10^−9^	-
hexavalent chromium	2.89 × 10^−4^	3.72 × 10^−8^	-

**Table 9:** Results of grouping and weighing environmental impacts for compounds affecting human health in the life cycle of a car tire.

Substance	Production, Pt	Use, Pt	Recycling, Pt
sulfur oxide	1.33 × 10^−2^	1.58 × 10^1^	−4.07 × 10^−3^
carbon dioxide	2.77 × 10^−2^	1.24 × 10^1^	−1.03 × 10^−2^
nitrogen dioxide	9.12 × 10^−3^	1.00 × 10^1^	-
dust particles, SPM	-	2.49 × 10^0^	-
sulfur dioxide	2.00 × 10^0^	5.83 × 10^−3^	-
carbon dioxide, fossil	1.69 × 10^0^	6.60 × 10^−3^	-
dust particles, <2.5 µm	1.48 × 10^0^	4.40 × 10^−3^	-
arsenic, ions	1.35 × 10^0^	7.79 × 10^−4^	−4.62 × 10^−3^
nitric oxide	1.31 × 10^0^	1.16 × 10^−2^	−1.68 × 10^−2^
dust particles, >2.5 µm, and <10 µm	1.67 × 10^−1^	3.99 × 10^−3^	-
cadmium, ions	1.15 × 10^−1^	9.28 × 10^−5^	−1.35 × 10^−4^
methane, fossil	8.79 × 10^−2^	4.94 × 10^−4^	-
hydrocarbons, unspecified	1.01 × 10^−5^	1.71 × 10^−2^	2.47 × 10^−6^
carbon dioxide, biogenic	1.63 × 10^−2^	3.35 × 10^−5^	-
arsenic	1.55 × 10^−2^	7.59 × 10^−8^	-
cadmium	1.31 × 10^−2^	2.03 × 10^−7^	−3.05 × 10^−4^
nitrous oxide	4.54 × 10^−3^	6.46 × 10^−7^	8.53 × 10^−6^
carbon monoxide	-	3.08 × 10^−3^	-
carbon dioxide in the air	−2.26 × 10^−2^	−1.75 ×10^−7^	-

**Table 10:** Results of grouping and weighing environmental impact for processes affecting the depletion of raw fossil materials throughout the life cycle of a car tire.

Substance	Production, Pt	Use, Pt	Recycling, Pt
natural gas, 42.7 MJ/kg, fossil	2.65 × 10^−3^	9.77 × 10^1^	-
natural gas, fossil gas	1.12 × 10^0^	7.08 × 10^−2^	-
crude oil, fossil	1.11 × 10^0^	6.44 × 10^−2^	-
hard coal, fossil coal	4.23 × 10^−1^	1.96 × 10^−4^	-
crude oil, 42.6 MJ/kg, fossil	2.96 × 10^−1^	-	−1.69 × 10^−2^
natural gas from waste gases generated in coal mining processes/m^3^	1.34 × 10^−1^	4.35 × 10^−7^	-
natural gas, 30.3 MJ/kg, fossil	6.69 × 10^−2^	-	-
natural gas, 35 MJ/m^3^, fossil	3.21 × 10^−2^	-	7.44 × 10^−3^
nickel, 1.98% in silicates, 1.04% in ore, fossil	5.18 × 10^−3^	7.75 × 10^−7^	-
copper, 2.19% in sulfides, Cu 1.83% and Mo 8.2E-3% in ore, fossil	1.62 × 10^−3^	1.07 × 10^−7^	-
iron, fossil	1.70 × 10^−9^	-	−1.70 × 10^−3^
natural gas, raw material, 35 MJ/m^3^, fossil	-	-	−4.28 × 10^−2^
natural gas, 36.6 MJ/m^3^, fossil	-	-	−4.12 × 10^−2^
crude oil, raw material, 41 MJ/kg, fossil	-	-	−4.35 × 10^−2^

**Table 11:** Assessment of energy consumption throughout the stages of the life cycle of a car tire using the CED method: characterization results.

Energy Raw Materials	Production, MJ Eq	Use, MJ Eq	Recycling, MJ Eq
non-renewable: Fossil fuels	4191.312	28,543.028	−50.043
non-renewable: Nuclear energy	69.612	1.090	16.128
renewable: Biomass	44.696	0.026	0.000
renewable: Wind, solar radiation, geothermal energy	1.684	2.20E-05	0.000
renewable: Water	23.519	0.101	1.878
**Sum**	**4330.824**	**28,544.245**	**−32.038**

**Table 12:** Assessment of greenhouse gas emissions at stages of the life cycle of a car tire using the Intergovernmental Panel on Climate Change (IPCC) method.

Production, Kg CO_2_ Eq	Use, Kg CO_2_ Eq	Recycling, Kg CO_2_ Eq
333,723	2,265,301	−2116

## Supplementary Materials

The following are available online at https://www.mdpi.com/1996-1944/12/24/4177/s1, Figure S1: Carcinogenic substances contribution in the impact category “carcinogens” in the life cycle stages of a car tire, Figure S2: Organic substances contribution in the impact category “respiratory organics” in the life cycle stages of a car tire, Figure S3: Ionizing substances contribution in the impact category “radiation” in the life cycle stages of a car tire, Figure S4: Compounds causing ozone layer depletion contribution in the impact category “ozone layer” in the life cycle stages of a car tire, Figure S5: Ecotoxic compounds contribution in the impact category “ecotoxicity” in the life cycle stages of a car tire, Figure S6: Compounds causing acidification/eutrophication contribution in the impact category “acidification/eutrophication” in the life cycle stages of a car tire, Figure S7: Minerals extraction contribution in the impact category “minerals” in the life cycle stages of a car tire.
