# Supplementary material for: Assessment of the Environmental Impact of a Car Tire throughout Its Lifecycle Using the LCA Method
Source: Materials (Basel). 2019 Dec 12;12(24):4177. doi: 10.3390/ma12244177 (PMC6947500; doi:10.3390/ma12244177)
Supplement: Supplementary file 1 [file materials-12-04177-s001.pdf]

materials

Supplementary

Assessment of the Environmental Impact of a Car Tire throughout Its Lifecycle Using the LCA Method

Katarzyna Piotrowska [1], Weronika Kruszelnicka [2,*], Patrycja Bałdowska-Witos [2], Robert Kasner [2], Jacek Rudnicki [3], Andrzej Tomporowski [2], Józef Flizikowski [2] and Marek Opielak [1]

[1] Faculty of Mechanical Engineering, Lublin University of Technology, 20-618 Lublin, Poland; k.piotrowska@pollub.pl (K.P.); m.opielak@pollub.pl (M.O.)
[2] Department of Technical Systems Engineering, Faculty of Mechanical Engineering, University of Science and Technology in Bydgoszcz, 85-796 Bydgoszcz, Poland; patrycja.baldowska-witos@utp.edu.pl (P.B.-W.); robert.kasner@gmail.com (R.K.); a.tomporowski@utp.edu.pl (A.T.); fliz@utp.edu.pl (J.F.)
[3] Faculty of Ocean Engineering And Ship Technology, Gdańsk University of Technology, 80-980 Gdańsk, Poland; jacekrud@pg.edu.pl
* Correspondence: weronika.kruszelnicka@utp.edu.pl

Received: 23 September 2019; Accepted: 10 December 2019; Published: date

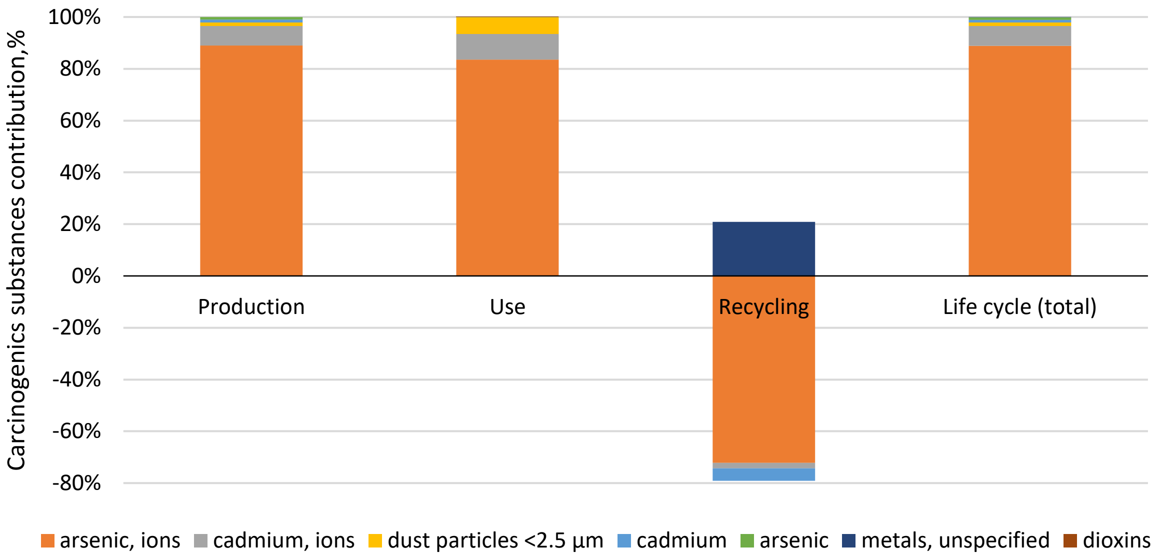

Figure S1. Carcinogenic substances contribution in the impact category "carcinogens" in the life cycle stages of a car tire.

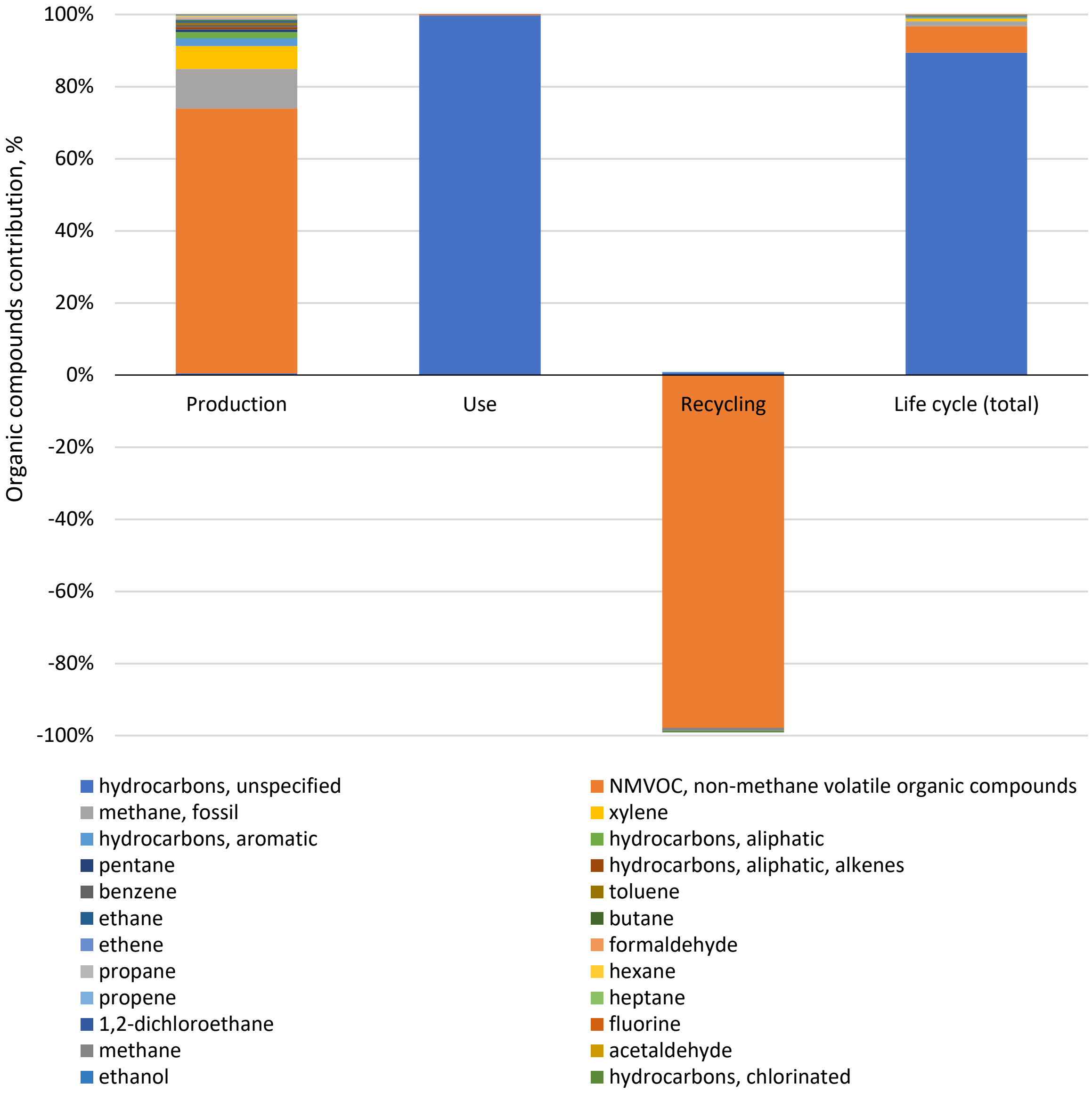

Figure S2. Organic substances contribution in the impact category "respiratory organics" in the life cycle stages of a car tire.

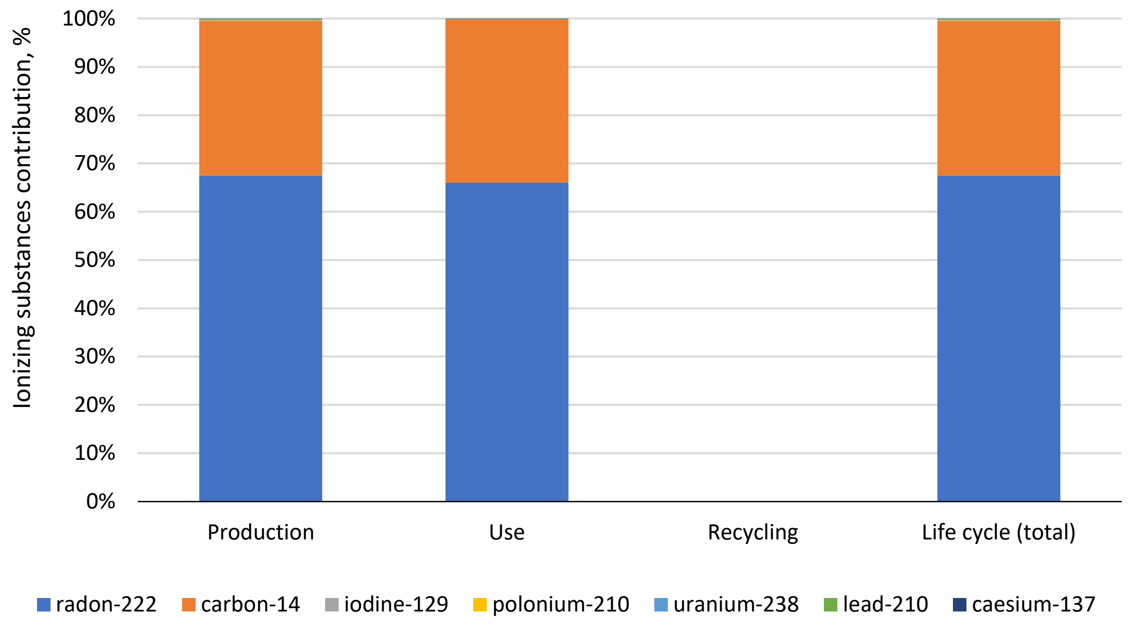

Figure S3. Ionizing substances contribution in the impact category "radiation" in the life cycle stages of a car tire.

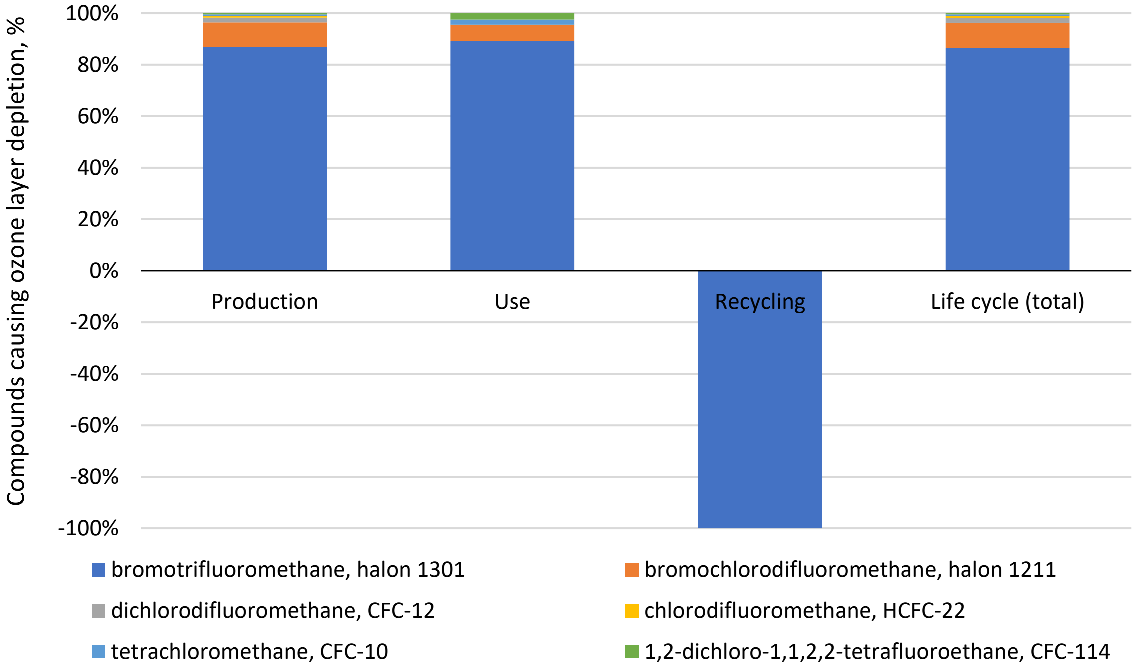

Figure S4. Compounds causing ozone layer depletion contribution in the impact category "ozone layer" in the life cycle stages of a car tire.

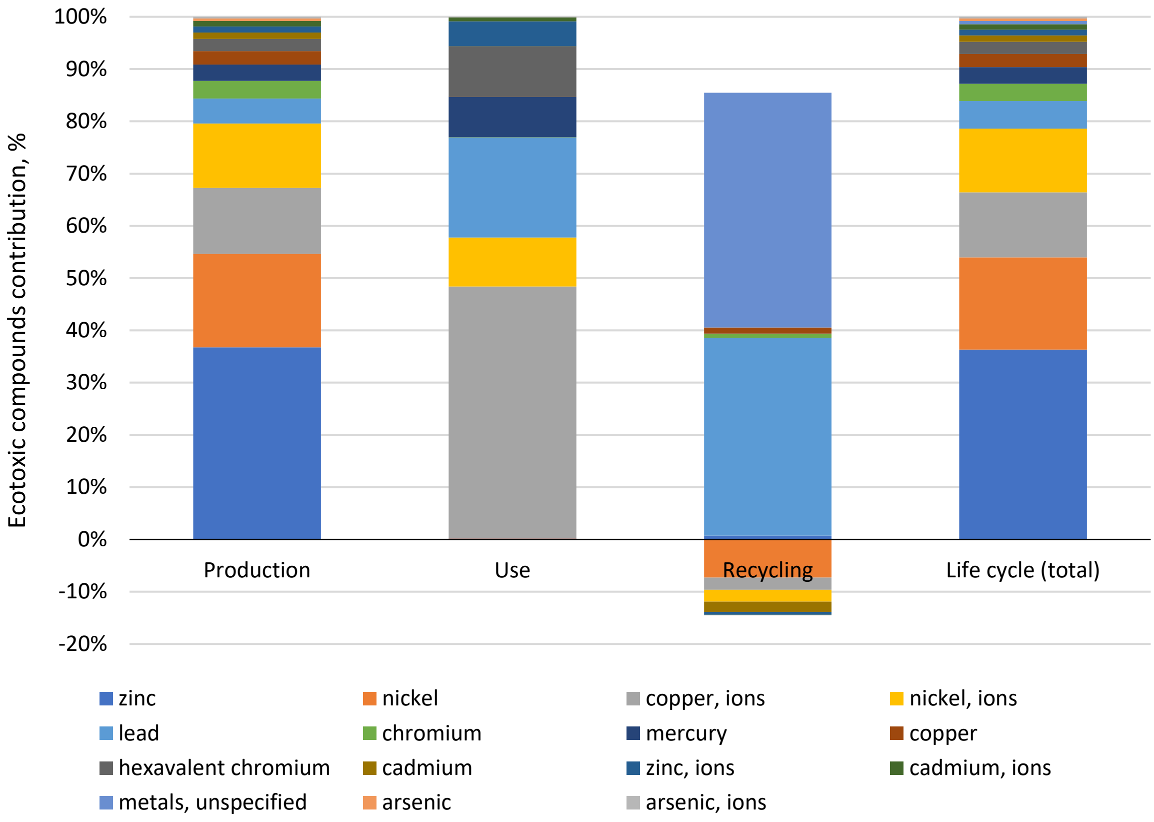

Figure S5. Ecotoxic compounds contribution in the impact category "ecotoxicity" in the life cycle stages of a car tire.

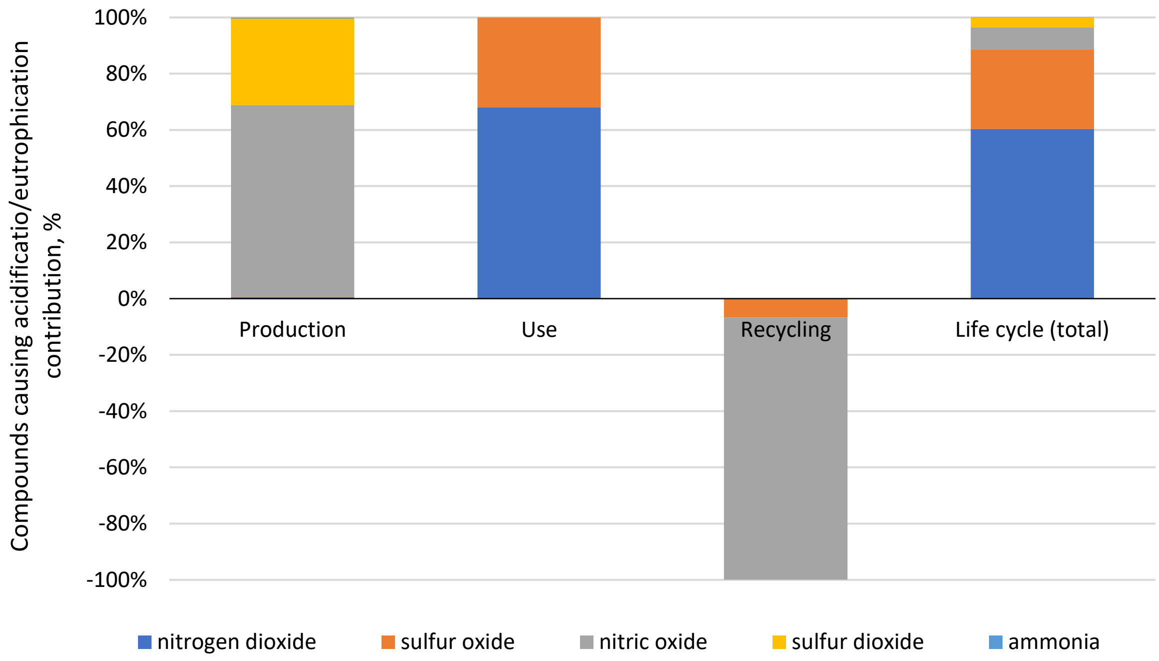

Figure S6. Compounds causing acidification/eutrophication contribution in the impact category "acidification/eutrophication" in the life cycle stages of a car tire.

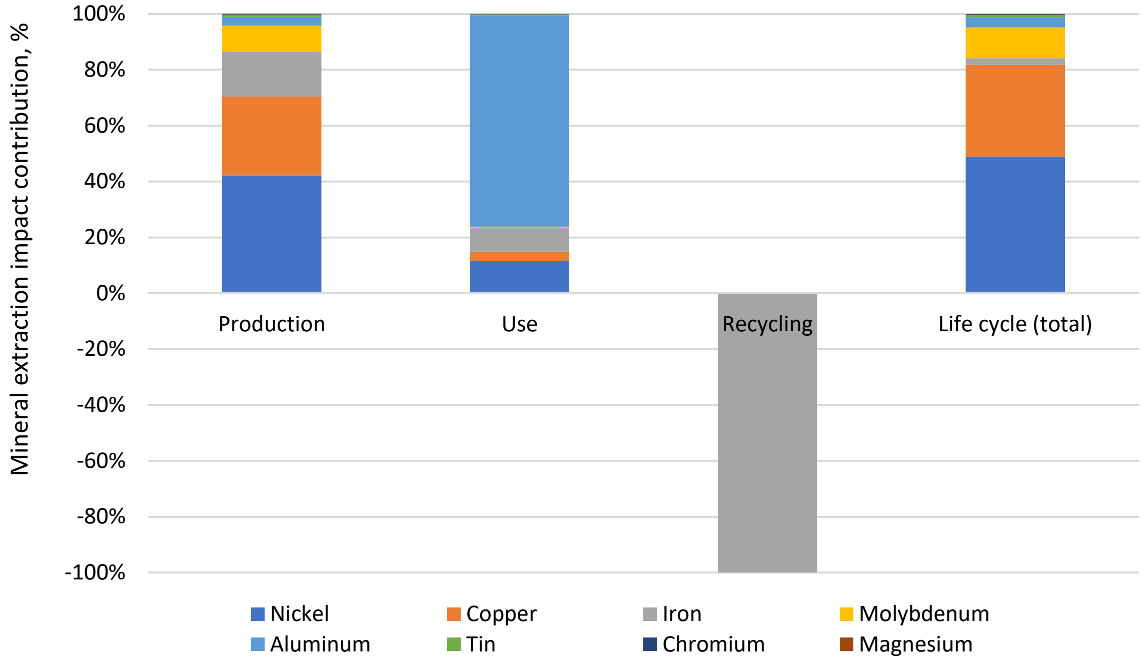

Figure S7. Minerals extraction contribution in the impact category "minerals" in the life cycle stages of a car tire.

© 2019 by the authors. Submitted for possible open access publication under the terms and conditions of the Creative Commons Attribution (CC BY) license (http://creativecommons.org/licenses/by/4.0/).
